# Gastric Duplication Cyst: A Rare Congenital Disease Often Misdiagnosed in Adults

**DOI:** 10.1155/2013/850967

**Published:** 2013-02-12

**Authors:** Jessica Falleti, Elena Vigliar, Pio Zeppa, Pietro Schettino, Vincenzo Napolitano, Maria D'Armiento

**Affiliations:** ^1^Section of Pathology, Department of Biomedical Sciences, University of Naples “Federico II,” Via Pansini 5, 80131 Naples, Italy; ^2^Division of Surgical Oncology, “F. Magrassi-A. Lanzara” Department of Clinical and Experimental Medicine and Surgery, Second University of Naples School of Medicine, c/o II Policlinico, Edificio 17, Via Pansini 5, 80131 Naples, Italy

## Abstract

Gastrointestinal duplication is a rare congenital disease which affected more commonly the ileum, while the stomach is rarely involved. Generally diagnosed in paediatric or young age, it could be difficult to suspect a gastrointestinal duplication in adults. Herein, we report a 55-year-old male with a gastric duplication cyst found on routinely checkup for chronic hepatitis and first misdiagnosed as a gastrointestinal stromal tumor (GIST); we also discuss its embryology.

## 1. Introduction

Gastrointestinal duplication is a rare congenital disease defined as a spherical hallow structure with a smooth muscle coat, lined by a mucus membrane and attached to any part of the gastrointestinal tract from the base of the tongue to the anus; because these malformations are formed before differentiation of the lining epithelium, they are named for the organs with which they are associated [1]. Most common involved sites are ileum followed, in order of frequency, by oesophagus, jejunum, stomach, and colon [2]. Gastric duplication cysts are very uncommon with a reported incidence of 4%–8% among all gastrointestinal duplication cysts [3].

Herein, we report a gastric duplication cyst in a 55-year-old man first misdiagnosed as a gastrointestinal stromal tumor (GIST) and discuss its embryological origin.

## 2. Case Presentation

A 55-year-old man suffering from B type chronic hepatitis treated with antiretroviral drugs was found to have a hypoechoic round-shaped mass sized 4.7 cm, with regular margins, during an abdominal ultrasonography performed for scheduled checkup. The mass was located between the left liver lobe and the anterior surface of the pancreatic body.

The patient did not complain of any symptoms about gastrointestinal tract.

**Figure 1:**
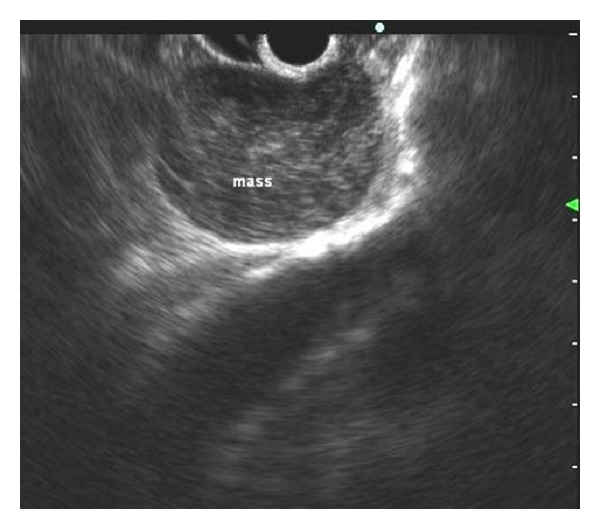
Endoscopic ultrasonography showing a hypoechoic mass with a slightly heterogeneous internal echo and regular margins located just below the gastroesophageal junction.

To better evaluate the mass, patient underwent MR imaging that confirmed the presence of a cystic mass with complex content located forward to the gastroesophageal junction. A subsequently endoscopic ultrasonography showed a hypoechoic mass with a slightly heterogeneous internal echo and regular margins located just below the gastroesophageal junction; the lesion measured about 4.5 cm and seemed to be contiguous to the fourth wall layer (muscularis propria) (Figure 1).

**Figure 2:**
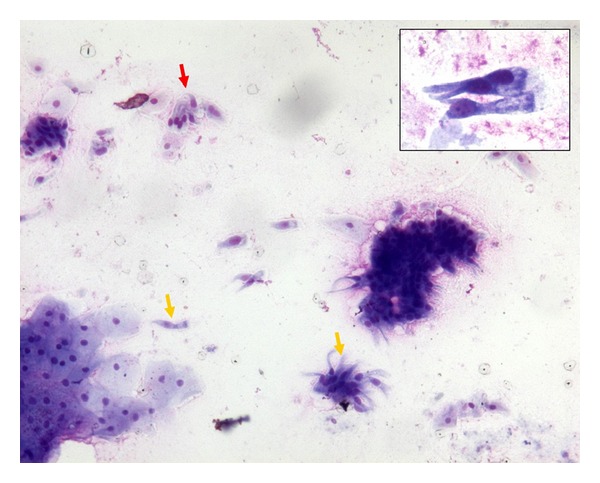
Cytological smears showed, in a mucoid background, scattered histiocytes, gastric and oesophageal mucosal cells, and few groups of ciliated columnar cells (MGG, 200x; inset 400x).

On the basis of a diagnosis suspicious for GIST involving the upper part of the gastric wall, a diagnostic confirmation through an EUS-guided fine needle aspiration with a 22 G needle was taken; it was aspirated mucoid material. Cytological smears showed, in a mucoid background, scattered histiocytes, gastric and oesophageal mucosal cells, and few groups of ciliated columnar cells (Figure 2).

However, a surgical excision was performed.

Resected specimen measured about 5 × 3 × 3 cm and was sutured on one site with mechanical clips; on cut-section, it was cystic with smooth wall and filled with mucus-like fluid.

**Figure 3:**
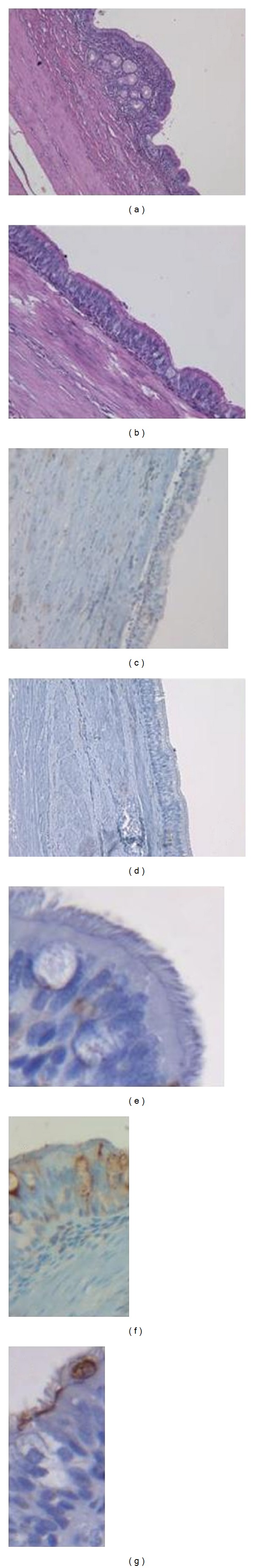
Cyst's wall was focally lined by gastric foveolar epithelium with cardial glands ((a) H/E, 200x), while most of the cyst was lined by a pseudostratified epithelium with ciliated cells ((b) H/E, 200x). Pseudostratified columnar ciliated epithelium was negative for CK20 ((c) 200x), TTF-1 ((d) 200x), and MUC5AC ((e) 400x) while expressed CEA ((f) 200x) and MUC1 ((g) 400x).

Histologically, the wall consisted of mucosa, subepithelial connective tissue, a layer of smooth muscle, and an outer fibrous capsule. Focally, the mucosa was lined by gastric foveolar epithelium with cardial glands (Figure 3(a)). Most of the cyst was lined by a pseudostratified epithelium with ciliated cells (Figure 3(b)). No intestinal-type epithelium was present. We tested expression of CK7, CK20, CEA, TTF-1, MUC1, and MUC5AC; on ciliated epithelium, CK20, TTF-1, and MUC5AC resulted negative (Figures 3(c), 3(d), and 3(e)), while CK7, CEA, and MUC1 appeared to be expressed (Figures 3(f) and 3(g)).

A diagnosis of gastric duplication cyst lined by pseudostratified columnar ciliated epithelium was made.

## 3. Discussion

Intestinal duplications or enterogenous cysts are spherical or tubular structures possessing the basic pattern of the enteric wall; they can be located in any part of the alimentary tract even if usually duplications are intraabdominal with the small intestine being the most commonly involved, particularly the distal ileum.

About foregut duplications, those associated with oesophagus are more common, while gastric duplications are rare and usually located on the greater curvature being twice more common in female. A variety of mechanisms have been invoked about their origin like failure of recanalization of the bowel lumen following the so-called solid-epithelial phase of the intestinal development, persistence of epithelial outpunching described in embryonic intestine, intestinal ischemia in early intrauterine life, impaired separation of the notochord from intestinal endoderm, and the formation of neurenteric bands with embryonic growth, producing traction diverticula [4].

Gastric duplication cysts are usually diagnosed in a younger age due to their position and mass effect; in adults, diagnosis may be difficult because of the wide range of symptoms and signs reported [1]. 

Moreover, radiological findings can overlap with some other pathologies like in our case in which it was misdiagnosed, on MR and endoscopic ultrasonography, with a cystic GIST. However, when it can be performed, cytology could aid in the differential diagnosis; in this case, it did not show malignant cells but a mucoid background containing normal cells.

More commonly, gastrointestinal duplication cysts are lined by typical gastric mucosa even if in some cases it can be a ciliated pseudostratified epithelium like in our case. The presence of respiratory epithelium could suggest an explanation to its origin because some authors have demonstrated expression of TTF-1 and surfactant [5].

Early in the 3rd week, the respiratory diverticulum appears along the ventral wall of the pharyngeal gut. In this phase, the dorsal portion, which becomes the oesophagus, and the ventral portion, which becomes the trachea, are both lined by a pseudostratified columnar ciliated epithelium. Subsequently, the oesophageal mucosa undergoes squamous metaplasia [4]. 

Khoury and Rivera[5] described two cases of gastric duplication cyst with a pseudostratified columnar ciliated epithelium; both cysts expressed TTF-1 and surfactant. Based on the presence of ciliated epithelium and expression of TTF-1, the authors proposed that a branch coming off the respiratory diverticulum gives rise to the duplication cyst. We also tested the expression of TTF-1, MUC1, and MUC5AC in our case; the pseudostratified columnar ciliated epithelium did not express TTF-1 and MUC5AC, while MUC1 appeared to be positive. There are two possible explanations for this phenomenon. The expression of TTF-1 is not an early event in the respiratory diverticulum development, but it appears starting from 11 to 12 weeks' gestation in human fetuses [6]. Therefore, a duplication cyst formed before 11 weeks' gestation could not acquire the expression of TTF-1. On the other hand, studies on mouse embryos, that have examined the spatial pattern of TTF-1 expression, have shown positivity for TTF-1 confined to the ventral portion of foregut; no staining was detected in the epithelium of the dorsal wall, which becomes the oesophagus [7]. Therefore, a duplication cyst arising from the most dorsal portion of foregut could not express TTF-1.

The expression of MUC1 is explained since MUC1 is normally found on surface respiratory cells [8].

In our case two hypothesis are available about duplication cyst's origin: formation before 11 weeks' gestation or from the most dorsal portion of foregut, so further studies needed to better understanding the embryogenesis of intestinal duplication cysts. However, whatever could be the origin, it is important for the practitioner physician to know that this unusual embryological malformation can also occur in adult people, and it must be considered in the differential diagnosis of thoracoabdominal masses.
